# Multiple functions of the nonstructural protein 3D in picornavirus infection

**DOI:** 10.3389/fimmu.2024.1365521

**Published:** 2024-04-02

**Authors:** Chenxia Xu, Mingshu Wang, Anchun Cheng, Qiao Yang, Juan Huang, Xumin Ou, Di Sun, Yu He, Zhen Wu, Ying Wu, Shaqiu Zhang, Bin Tian, Xinxin Zhao, Mafeng Liu, Dekang Zhu, Renyong Jia, Shun Chen

**Affiliations:** ^1^ Engineering Research Center of Southwest Animal Disease Prevention and Control Technology, Ministry of Education of the People’s Republic of China, Chengdu, China; ^2^ Key Laboratory of Animal Disease and Human Health of Sichuan Province, Chengdu, China; ^3^ International Joint Research Center for Animal Disease Prevention and Control of Sichuan Province, Chengdu, China; ^4^ Institute of Veterinary Medicine and Immunology, Sichuan Agricultural University, Chengdu, China; ^5^ Research Center of Avian Disease, College of Veterinary Medicine, Sichuan Agricultural University, Chengdu, China

**Keywords:** picornavirus, 3D polymerase, virus replication, nuclear localization signal, interactions, innate immunity

## Abstract

3D polymerase, also known as RNA-dependent RNA polymerase, is encoded by all known picornaviruses, and their structures are highly conserved. In the process of picornavirus replication, 3D polymerase facilitates the assembly of replication complexes and directly catalyzes the synthesis of viral RNA. The nuclear localization signal carried by picornavirus 3D polymerase, combined with its ability to interact with other viral proteins, viral RNA and cellular proteins, indicate that its noncatalytic role is equally important in viral infections. Recent studies have shown that 3D polymerase has multiple effects on host cell biological functions, including inducing cell cycle arrest, regulating host cell translation, inducing autophagy, evading immune responses, and triggering inflammasome formation. Thus, 3D polymerase would be a very valuable target for the development of antiviral therapies. This review summarizes current studies on the structure of 3D polymerase and its regulation of host cell responses, thereby improving the understanding of picornavirus-mediated pathogenesis caused by 3D polymerase.

## 1 Introduction

Picornaviruses represent one of the largest virus groups and include several important human and animal pathogens, such as poliovirus (PV), coxsackievirus (CV), enterovirus (EV), rhinovirus (RV), encephalomyocarditis virus (EMCV), and foot-and-mouth disease virus (FMDV) (1). To date, the family *Picornaviridae* consists of 158 species grouped into 68 genera (as of March 2022), such as *Enterovirus*, *Hepatovirus*, *Cardiovirus* and *Aphthovirus* (2, 3).

**Figure 1:**
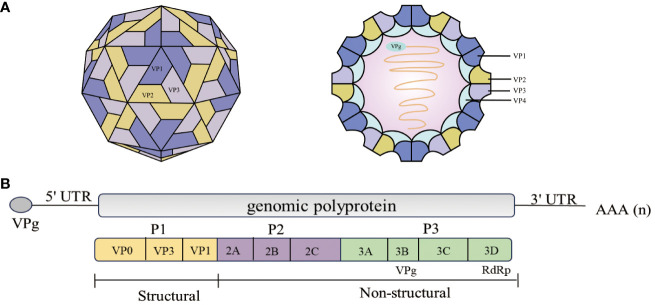
Schematic representation of the EV71 structure and genome structure of the virion. **(A)** Structure of the EV71 (6). EV71 is a small (circumference around 30 nm), non-enveloped, icosahedral particle that contains a single-stranded, positive-sense, polyadenylated virus RNA of approximately 7.4 kb (6). **(B)** The diagram demonstrates the EV71 genome structure. All the structural proteins are encoded by the P1 region (yellow) of the genome. The P2 (purple) and P3 (green) regions encode seven non-structural proteins—2A–2C and 3A–3D. The last part of the polyprotein is 3D^pol^, the RNA-dependent RNA polymerase that is active only upon cleavage of the 3C^pro^–3D^pol^ junction (7, 8).

The members of the *Picornaviridae* family are small, nonenveloped RNA viruses. The picornavirus virion has a symmetrical icosahedral spherical structure with an approximate diameter of 20-40 nm (4–6). The viral genome is a single-stranded, positive RNA strand approximately 6.7-10.1 kb in length that consists of an open reading frame (ORF), a highly structured 5′ untranslated region (5′ UTR), and a 3′ untranslated region (3′ UTR) with a [poly(A)] tail (**Figure 1**) (9). The viral genome-linked protein 3B (also known as VPg) is covalently bound to the 5′ end of the positive-sense RNA (10). The 5′ UTR harbors an internal ribosomal entry site (IRES) that recruits ribosomes and other host factors and mediates cap-independent translation (11, 12). The ORF initially encodes a single polyprotein that is co and posttranslationally cleaved by viral proteases to release the capsid proteins VP0, VP1, and VP3 and nonstructural proteins (2A, 2B, 2C, 3A, 3B, 3C, 3D), as well as some stable precursors, such as 3AB or 3CD, that are essential for the replication of viral RNA (13–15). Recently, a second ORF termed the upstream ORF (uORF) was identified in enteroviruses (16). Some genera of picornaviruses, such as *Aphthoviruses and Cardioviruses*, also have a leading conductor (L) protein at the N-terminus of the polyprotein (17). This review mainly focuses on 3D polymerase (3D^pol^).

**Figure 2:**
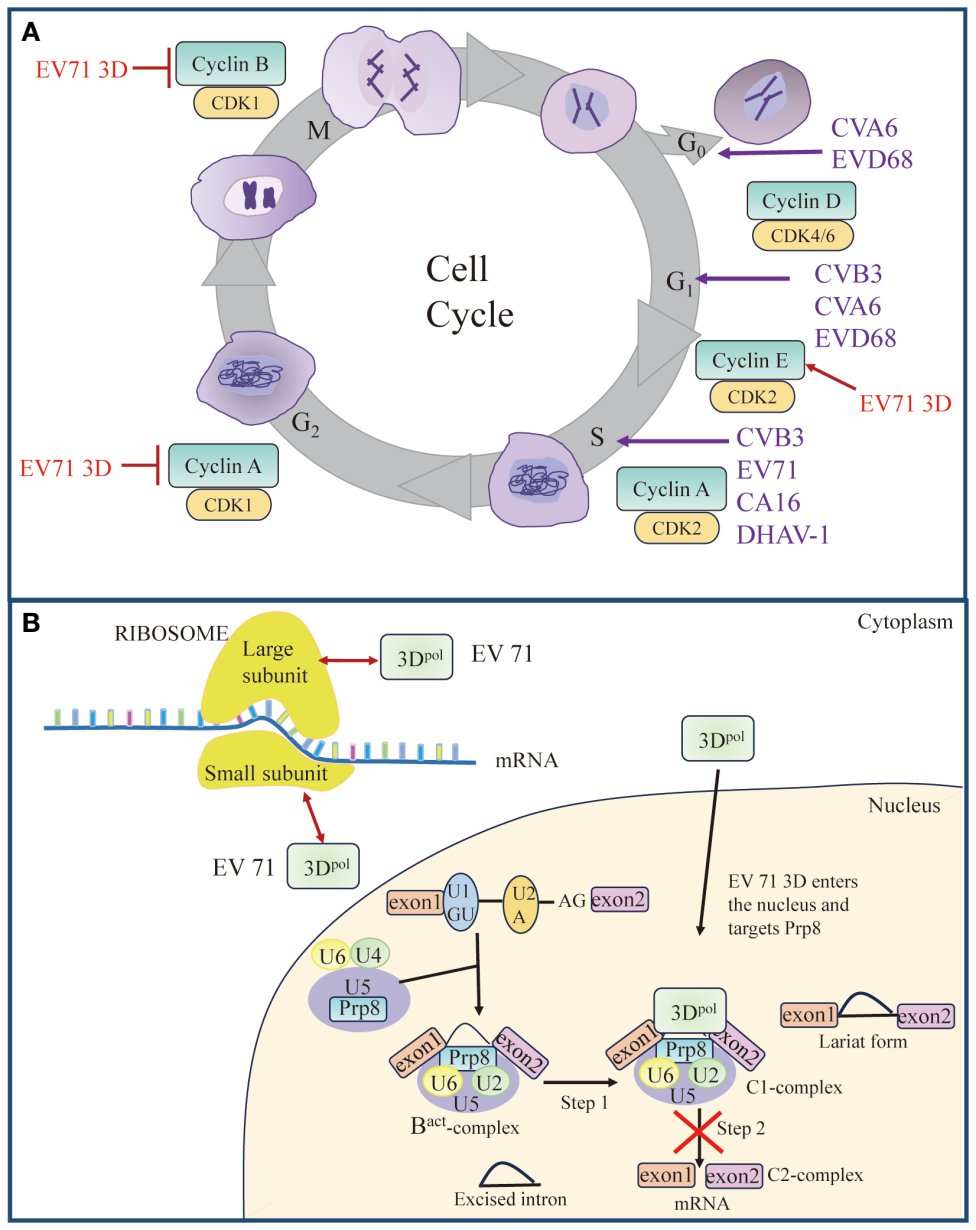
Roles of 3D^pol^ in cell cycle and cell translation. **(A)** Picornavirus 3D^pol^ induces cell cycle arrest. Red arrow represents “Upregulate”, and red vertical symbols represent “Inhibit”. **(B)** Schematic model of 3D^pol^-mediated effects on cellular translation. EV 71 3D^pol^ could enhance EV-A71 IRES-dependent translation as well as cap-dependent translation by interacting with small and large subunits of ribosomes. Partially, 3D^pol^ also enters the nucleus and interacts with the core splicing factor Prp8, which interferes with the function of Prp8 in the C1-complex. The interference of the Prp8 function inhibits the second step of the splicing process and results in the accumulation of the lariat form and a reduction in mRNA synthesis. Red bidirectional arrows represent “Interact”. The figure was modified from (24).

The picornavirus 3D^pol^, also known as RNA-dependent RNA polymerase (RdRp), is responsible for genome synthesis (18, 19). 3D^pol^ becomes active upon cleavage of the precursor 3CD protease (3CD^pro^) (7, 20). Previous studies have yielded a very good understanding of the 3D^pol^ structure and fundamental molecular mechanism for catalysis (21). The N-terminal region of 3D^pol^ acts as a nuclear localization signal (NLS), which is involved in nucleotide recognition and affects the incorporation of nucleotide analogs, suggesting the multifunctionality of the picornavirus polymerase domains (22). In addition, recent studies have revealed novel mechanisms for picornavirus invasion of host cells involving multiple previously undiscovered functions of 3D^pol^ that differ from its traditional role in viral replication. For example, EV71 3D^pol^ can enter the cellular nucleus through the NLS to associate with the core splicing factor pre-mRNA processing factor 8 (Prp8), affecting the normal function of Prp8 during the second catalytic splicing step, leading to the inhibition of pre-mRNA splicing, the accumulation of the lariat form, and a decrease in the resulting mRNA; or it can facilitate viral and host translation by forming complexes with small and large subunits of ribosomes (23, 24) (**Figure 2B**); 3D^pol^ also functions as an antagonist against the host innate immune response (25–27). In this review, we summarize the general structural features and functions of 3D^pol^ and discuss the role of 3D^pol^ in regulating virus−host interactions to promote viral replication.

## 2 Structural features of the picornavirus 3D^pol^

### 2.1 Overall 3D^pol^ structure

**Figure 3:**
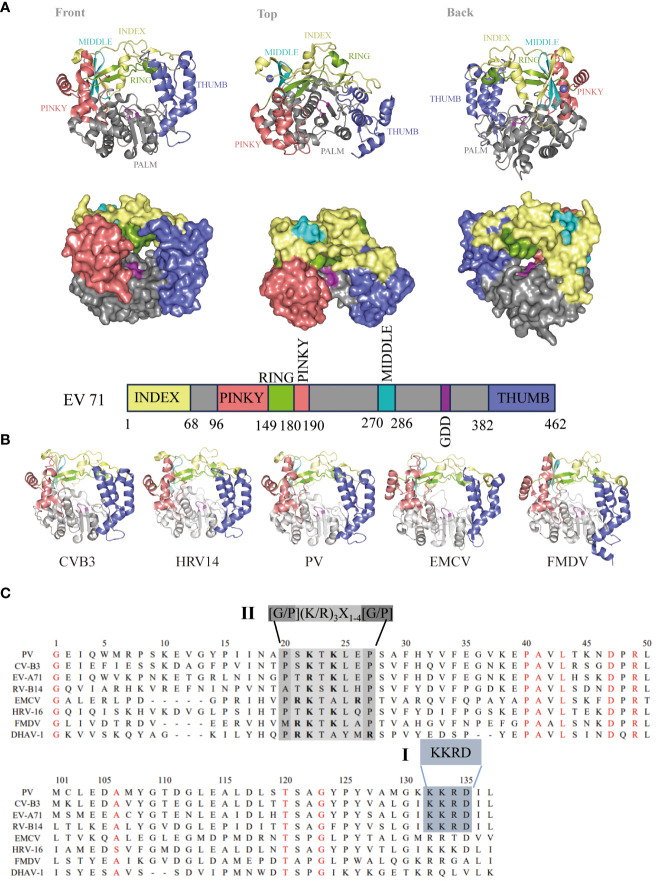
Genome and structure of picornavirus 3D^pol^. **(A)** Cartoon and surface representations of EV71 3D^pol^ (Protein Data Bank: 3N6L) in three different orientations. The structure resembles a cupped right hand composed of palm, fingers, and thumb domains; an index finger (residues 1-68) in yellow; a middle finger (270–286) in canyon; a ring finger (150–179) in green; a pinky finger (96–149, 180–190) in pink; a palm (191–269, 287–381) in gray; a thumb (382–462) in purple; and a GDD active site (328–320) in magenta. Bar representation of the 3D^pol^ sequence colored according to the structural elements are shown in **(A)**. **(B)** 3D^pol^ structures of CVB3 (PDB: 3DDK), HRV14 (PDB: 1XR5), PV (PDB: 2ILY), EMCV (PDB: 4NYZ) and FMDV (PDB: 1U09) are shown, and all of them exhibit a very high degree of structural homology. **(C)** Localization of the putative nuclear localization sequence (NLS) on picornavirus 3D sequences. Alignment of the amino acid sequences corresponding to the 3D amino termini from PV, CV, EV, RV, EMCV and DHAV-1. The single basic NLS KKRD (I) and the consensus NLS found in several yeast ribosomal proteins, G/P(KR)_3_X_1-4_[G/P] (II), are indicated (30).

Following the first report of the complete crystal structure of PV 3D^pol^ in 2004 (8), crystal structures of 3D^pol^ from HRV (28) and FMDV (29) were reported in succession. To date, there are several viral RdRp structures in the Protein Data Bank (PDB, www.wwpdb.org) related to different picornaviruses, including PV, CVB3, EV71, HRV, EMCV, and FMDV (21). Like other DNA and RNA polymerases, the crystal structure of the picornavirus 3D^pol^ resembles a cupped right hand, with three defined subdomains, termed the thumb, fingers and palm (**Figures 3A, B**) (8, 28, 29, 31–33). The finger domain can be further divided into distinct substructures that are sometimes referred to by the anatomical analogy of the index, ring, middle, and pinky domains. The thumb domain interacts with the finger domain to “close” the hand and envelops the active site, forming an NTP entry channel behind the RdRp (8, 28, 29, 31–33). The palm subdomain, consisting of two α spiral and five β-barrel domains (29), is the catalytic region of 3D^pol^ with a GDD-3 amino acid active site shared by all RdRps, and can bind Mg^2+^ and locate NTP substrates (34). In addition, 3D^pol^ contains seven conserved motifs (A to G) that play key roles in rNTP substrate recognition, template/primer binding and catalysis (35). Currently, available data provides high-resolution pictures for a range of conformational states associated to template and primer recognition, VPg uridylylation, rNTP recognition and binding, catalysis and chain translocation (36). These structural information provide insights into both initiation of RNA synthesis and the replication elongation processes in picornavirus (37, 38). The increased understanding of polymerase structure could help explore possible ways of vaccine development.

### 2.2 Nuclear localization signal

The picornavirus 3D^pol^ primarily replicates in the host cytoplasm, but 3D^pol^/3CD^pro^ can enter the nucleus in virus-infected cells (39–41). Previous studies have shown that PV 3D^pol^ and 3CD^pro^ enter the nucleus through a single basic type of nuclear localization signal (NLS), KKKRD, which spans 125–129 amino acids (aa) within 3D^pol^ (40, 41). The putative NLS is partially contained within the KKRD sequence (126–129 aa), which is typical among all known picornaviral 3D^pol^ (40, 42). However, these motifs are not completely reiterated in the other members of this family. In contrast, a NLS (^15^PRKTALRP^22^ in EMCV), similar to that in many yeast ribosomal proteins (43), was identified near the N-terminus of the EMCV 3D^pol^ sequence (44). An NLS similar to that of EMCV was also found in the 3D^pol^ of HRV16, FMDV and duck hepatitis A virus type 1 (DHAV-1) (30, 45, 46). By comparing the 3D^pol^ amino acid sequences, we found that NLSs within 3D^pol^ were mainly divided into two types: single basic NLSs, KKRDs, and other NLSs, which are similar to those found in several yeast ribosomal proteins, G/P(KR)_3_X_1-4_[G/P] (**Figure 3C**). Because studies have shown that the virus-encoded 3C protease (3C^pro^) cleaves transcription factors at glutamine–glycine sites and is directly responsible for host cell transcription shut-off, it is likely that 3C^pro^ must enter the nucleus of infected cells as is or in the form of a precursor (47). These data suggest that the NLS present within 3D^pol^ plays a role in the nuclear entry of precursor 3CD or 3BCD. This mechanism may be a common feature of picornavirus infections (48–50).

NLS sequences have functions other than facilitating the entry of viral proteins into the nucleus. The ^16^MRKTKLAPT^24^ sequence in 3D^pol^ of FMDV was identified as an NLS, and substitutions at the K18 or K20 residues resulted in two conformational changes that reduced 3D^pol^ binding to RNA (22); moreover, K18 and K20 were demonstrated to be essential for virus proliferation (45). In addition, the T19 and L21 residues are important for maintaining the fidelity of FMDV RdRps and ensuring faithful replication of the FMDV genome (51). Thus, the role of this class of NLS motifs in picornavirus viral polymerases needs to be revisited.

### 2.3 RNA structure in the 3D^pol^-coding region

The genomes of RNA viruses often contain RNA structures that are crucial for translation and RNA replication and may play additional roles during the viral replication cycle (52–55). For picornaviruses, within the ORF, several RNA structures have been identified. The cis-acting replication element in the 2C coding region (2C-CRE), which acts as a template for uridylylation of the VPg (3B) protein (56, 57), and an RNA structure carried in the 3C^pro^ ORF that potently inhibits the endonuclease activity of RNase L (an antiviral endoribonuclease) have been identified (58, 59). In addition, in the PV genome, two stem loops (referred to as loops α and β) within the coding region of 3D^pol^ that are important for proper RNA synthesis during viral infection have been identified (54, 60). Previous studies have further shown the existence of a novel functional interaction between these RNA structures in the 3D^pol^-coding region and the viral protein (s) 3C^pro^ and/or its precursor 3CD^pro^ (54). Three of the RNA structures (ORF-SL51, ORF-SL52, and ORF-SL53) within the coding region of FMDV 3D^pol^ have also been identified, and they are critical for efficient replication of the FMDV replicon (61). Thus, the RNA structures formed by those genomic regions may play a functional role in the picornavirus replication cycle.

## 3 Posttranslational modifications of 3D^pol^

**Figure 4:**
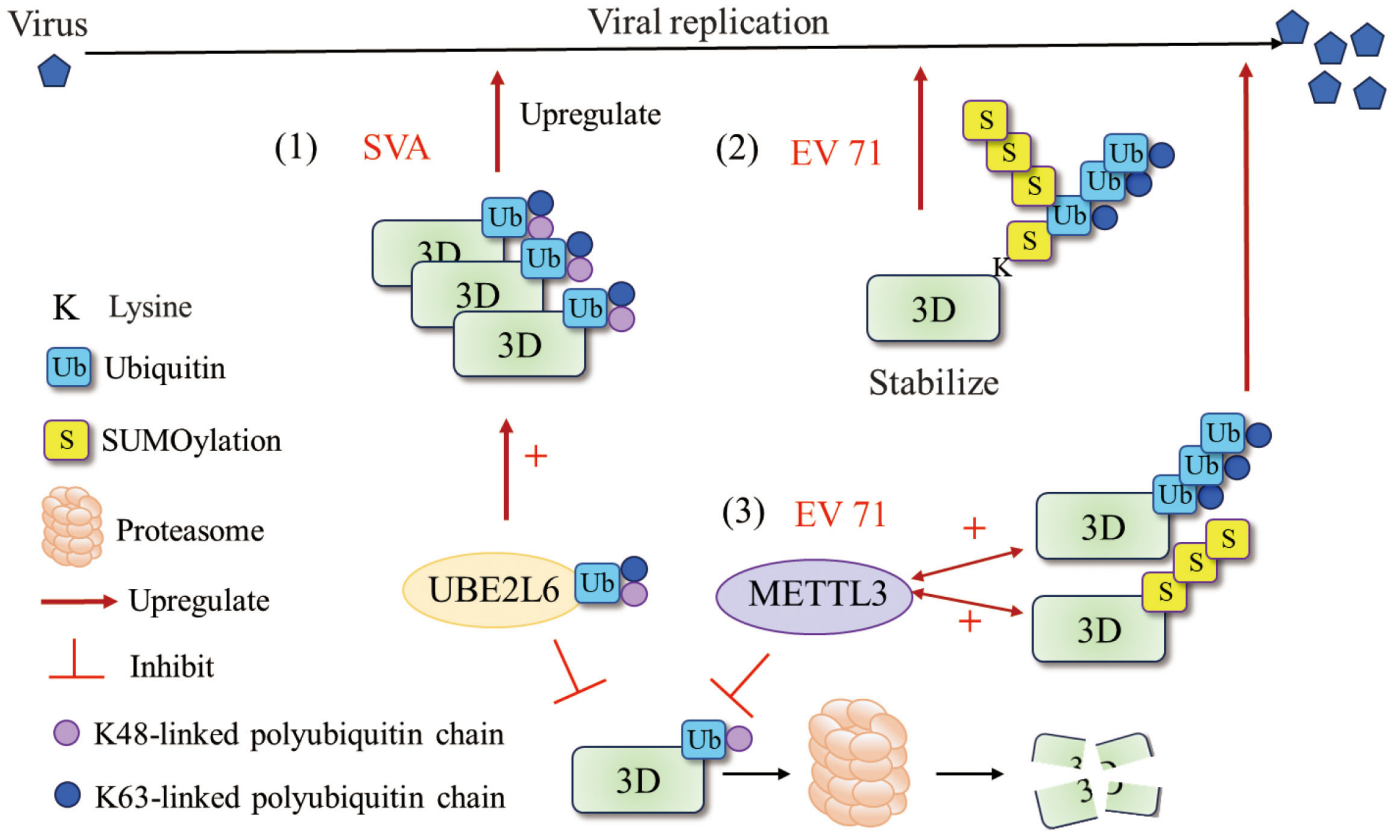
Ubiquitination and SUMOylation of 3D^pol^. (1) UBE2L6 interacted with SVA 3D^pol^ and mediated K48/K63 chains to improve the stability of 3D^pol^ (73). (2) EV71 exploit the cross talk of SUMOylation and ubiquitination to stabilize the 3D^pol^ and enhance viral replication. SUMOylation and ubiquitination may share the same lysine residues and that 3D^pol^ was ubiquitinated in a SUMO-dependent manner (74). (3) METTL3 interacted with EV 71 3D^pol^ and increased K63-linked ubiquitination and SUMOylation of the 3D^pol^ that boosted viral replication (75). Figure adapted from (73).

Ubiquitination and SUMOylation are widely studied posttranslational modifications (PTMs) that play critical roles in diverse biological processes (62, 63). The ubiquitin–proteasome system (UPS) also plays an important role in the different steps of the viral life cycle (64–66). The mechanisms by which the UPS regulates viral infection include the degradation of intracellular proteins or excess viral proteins and the modulation of viral protein function through ubiquitin-mediated modification or direct encoding of ubiquitin-related enzymes (67). An increasing number of studies have suggested that various viruses evolve different mechanisms to utilize or manipulate the host UPS for their own benefit (68–71). For picornaviruses, studies have shown that the UPS may regulate CVB3 replication through ubiquitinating viral 3D^pol^, which is essential for initiating viral RNA replication (72). In addition, Senecavirus A (SVA) 3D^pol^ is ubiquitinated by UBE2L6, an E2 ubiquitin-conjugating enzyme, and this ubiquitination serves to inhibit the degradation of 3D^pol^, thereby facilitating SVA infection (73) (**Figure 4**). Normally, the interplay between SUMOylation and ubiquitination often involves the stability of the target protein (76–78). EV71 3D^pol^ was modified by small ubiquitin-like modifier 1 (SUMO-1) both during infection and *in vitro*, and 3D^pol^ was ubiquitinated in a SUMO-dependent manner to enhance the stability of the viral polymerase (74). Moreover, residues K159 and L150/D151/L152 were found to be responsible for 3D^pol^ SUMOylation, and mutation of SUMOylation sites impaired 3D^pol^ activity and virus replication. Similarly, Hao et al. reported that the m^6^A methyltransferase METTL3 interacts with EV71 3D^pol^ and induces SUMOylation and ubiquitination of 3D^pol^, which boosts viral replication (75) (**Figure 4**). SUMOylation and ubiquitination of viral polymerases have been reported not only in picornaviruses but also in other viral families, such as nonstructural protein 5 (NS5) of dengue virus (79) and polymerase basic protein 1 (PB1) of influenza virus (80). Recent studies have characterized the alterations in UPS-dependent protein homeostasis during infection with CVB3 and demonstrated that the activity of the proteasome is exploited for the processing of viral precursor proteins (81). Furthermore, both the viral 3C^pro^ and the viral 3D^pol^ have been reported to be subjected to UPS-dependent proteolysis. This may be a strategy used by picornaviruses to maintain the proper balance of the expression levels of these two viral proteins to prevent premature cell death and ensure effective viral replication.

Studies have proposed that SUMOylation and ubiquitination at specific 3D^pol^ sites contribute to maintaining the cellular level of 3D^pol^ and that corresponding deSUMOylation and deubiquitination may be necessary for 3D^pol^ to restore polymerase activity since, when 3D^pol^ is responsible for RNA genome replication, it is free of SUMOylation (74). These findings imply that the 3D^pol^ of picornaviruses exploits host cell modifications for efficient replication, revealing potential targets for antiviral therapy.

## 4 Roles of 3D^pol^ in picornavirus replication

The picornavirus 3D^pol^ plays a critical role in viral genome replication by catalyzing different steps of viral genomic RNA replication, from primer synthesis (VPg-uridylylation) to viral RNA synthesis and polyadenylation of progeny genomic RNA. The first step in picornavirus genome replication is uridylylation of VPg. In this process, 3D^pol^ catalyzes the covalent attachment of two uridine monophosphate (UMP) molecules to the hydroxyl group of tyrosine 3 (Y3) of VPg and generates VPg-pUpU-OH, which serves as a primer to initiate the replication process (82–84). This process has been extensively studied in different members of the *Picornaviridae* family (37, 85–90), and detailed information can be found in this Review (90). Subsequently, 3D^pol^ catalyzes the synthesis of viral negative- and positive-sense RNA within the replication complex (RC) (91). In addition, to ensure genome integrity, a variable poly(A) tail is regenerated on the 3′ UTR end of newly synthesized viral RNAs during each round of viral replication (92–94). The length of poly(A) affects viral mRNA translation and RNA replication (95, 96). Previous studies have shown that virus replication can be severely impaired when the poly(A) tail is curtailed to 14 or 12 adenines or less (96–98). Alanine mutations in PV 3D^pol^ change the sizes of poly(A) tails in virion RNA, suggesting that 3D^pol^ is primarily responsible for the sizes of poly(A) tails (99–101).

Picornavirus infection induces the redistribution and rearrangement of cytoplasmic organelles to form membrane-bound structures that contribute to viral RNA replication; these structures are known as replicating organelles (ROs) (102–106). ROs may originate from Golgi membranes or the endoplasmic reticulum (ER) and contain host factors such as the lipid kinase PI4KB (also called PI4K IIIβ) as well as viral proteins, including 3A and 3D^pol^ (104, 107–109), which are thought to protect viral RNAs from RNase degradation or cellular RNA sensor detection (110–112). Increasing evidence suggests that proteins of picornaviruses hijack host factors involved in membrane trafficking and biosynthesis pathways to promote efficient viral genome replication (102, 113). Membrane-associated protein 3A (114) recruits PI4KB to the replication site through interaction with acyl-CoA binding domain containing 3 (ACBD3) (107, 108). PI4KB then catalyzes the formation of a phosphatidylinositol 4-phosphate (PI4P)-rich microenvironment that facilitates the recruitment of 3D^pol^ (102, 108).

**Figure 5:**
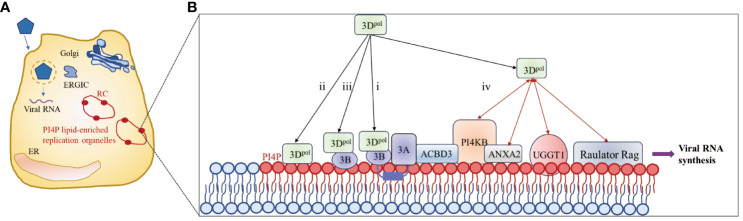
3D^pol^ is recruited to the surface of the lipid bilayer to promote RC formation. **(A)** Upon infection, synthesis of the 3A protein leads to remodeling of the Golgi/TGN and the endoplasmic reticulum-Golgi intermediate compartment (ERGIC) into replication organelles (ROs). PI4P lipids are indicated by red ovals. The enrichment of PI4P in these ROs promotes the binding of 3D^pol^ or 3CD to the membrane, which in turn facilitates the assembly of replication complexes (RCs) and the synthesis of viral RNA. **(B)** Four methods for recruiting 3D^pol^ to the surface of the lipid bilayer. The red bidirectional arrows represent interactions between viral and host proteins. The figure was modified from (105, 116).

Viral nonstructural proteins and their precursors, such as the 3A and 2BC proteins, contain hydrophobic regions that interact extensively with cell membranes and assemble to form RCs with cellular proteins and viral RNAs on the RO surface (112, 115). However, since picornavirus 3D^pol^ is a soluble protein with no obvious membrane-binding region, 3D^pol^ can be recruited to complex only by protein–protein or protein–RNA interactions (87). As shown in **Figure 5**, four methods for recruiting 3D^pol^ to the RO surface have been described in existing studies: i) 3AB, a small basic protein with biochemical properties similar to those of membrane proteins (117), interacts with 3D^pol^ through its VPg domain and recruits 3D^pol^ to the RC (117–121); ii) the PI4P lipid-rich microenvironment promotes the recruitment and stabilization of the 3D^pol^ membrane (102, 122); iii) negatively charged lipids cooperate with membrane-anchored 3B to recruit the 3D^pol^ enzyme (116); and (iv) host proteins recruit 3D^pol^ by interacting directly with 3D^pol^. Annexin A2 (ANXA2), which is localized on ROs, interacts with PI4KB, promotes the interaction of EV71 3D^pol^ with PI4KB and forms a higher-order protein complex with 3D^pol^ and PI4KB located in ROs (122). EV71 3D^pol^ interacts with host UDP-glucose glycoprotein glucosyltransferase 1 (UGGT1), a key ER protein involved in the unfolded protein response (UPR), to promote the formation of RCs on cellular membranes that enhance viral RNA synthesis (123). In addition, upon infection, the lysosomal tethered Ragulator-Rag complex promotes EV71/CVA16 replication by recruiting viral 3D^pol^ to the lysosomal surface through the interaction between 3D^pol^ and RagB (124). 3D^pol^, as part of a replication complex of 3A and several other viral proteins, subsequently initiates RNA synthesis at these membranes.

Interestingly, recent studies have shown that the CCT8, DBN1, IQGAP1 and ELMO2 proteins are involved in the regulation of cytoskeleton assembly and interact with EV71 3D^pol^, suggesting that viral 3D^pol^ may also play a role in cytoskeletal rearrangement during infection (23).

## 5 Regulation of host cell responses by 3D^pol^

**Figure 6:**
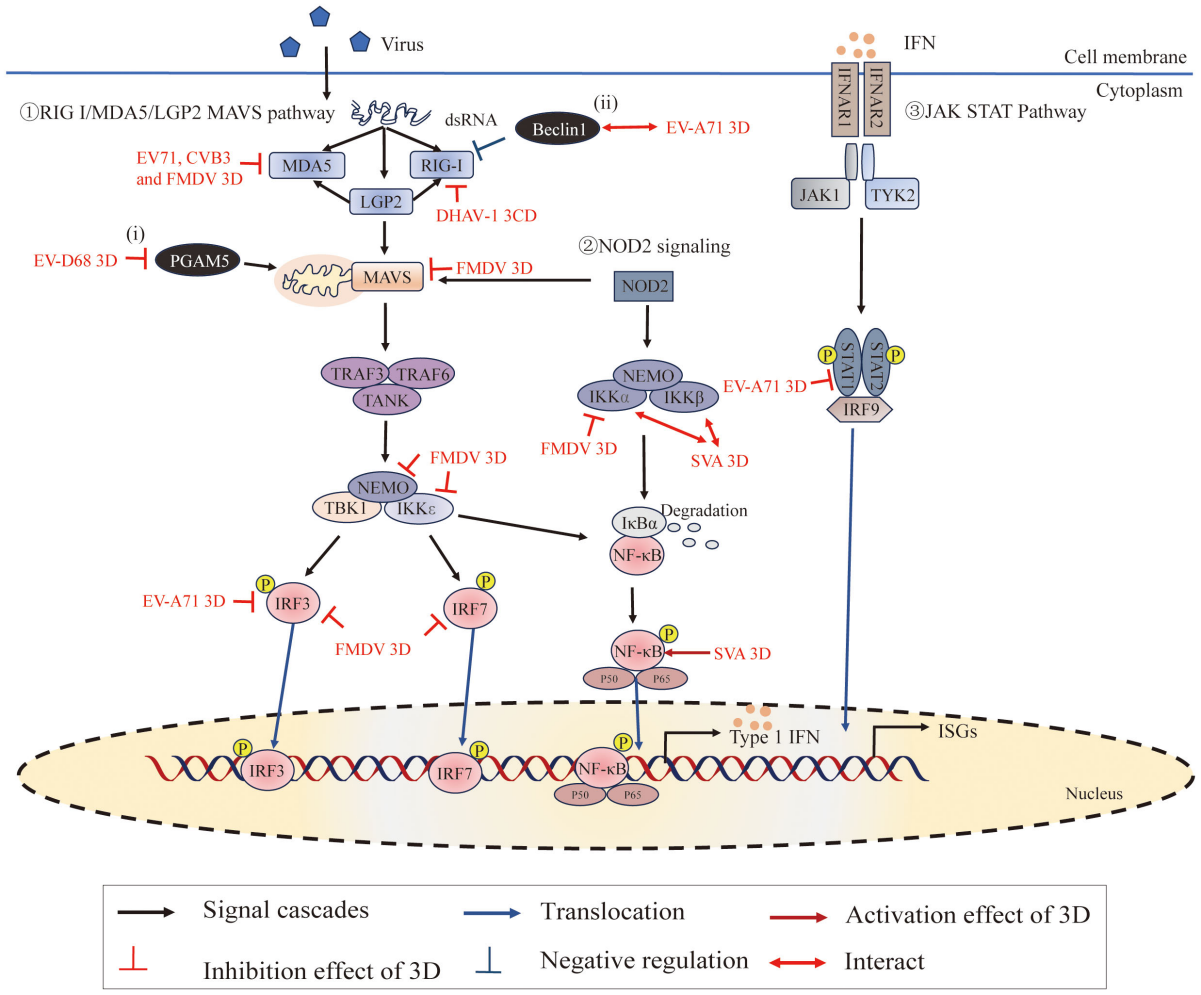
Roles of 3D^pol^ in subverting host innate immunity. ①The RIG-I/MDA5/LGP2-MAVS pathway recruits downstream adaptors, including tumor necrosis factor (TNF) receptor-associated factor 3 (TRAF3), TRAF6, and the TRAF family member-associated NF-κB activator (TANK), to directly induce the TBK1-IKKϵ-NEMO complex. These signaling cascades lead to the phosphorylation of interferon regulatory factors (IRFs) and NF-κB in the nucleus, where they promote the expression of interferons (IFNs), interferon-stimulated genes (ISGs) and proinflammatory cytokines (125). ②NOD2 signaling induces the activation of MAVS and the IKKα-IKKβ-NEMO complex. ③IFNs bind to IFN-α/β receptors (IFNARs), activating the Janus kinase-signal transducer and activator of transcription (JAK/STAT) pathway to amplify IFN production (126). Moreover, (i) PGAM5 affects mitochondrial morphology and affects the expression of MFN2, and MFN2 binds to MAVs to inhibit the RIG-I-like signaling pathway; (ii) Beclin-1 is a negative regulator of the RIG-I-MAVS-mediated IFN response. Figure adapted from (127).

**Figure 7:**
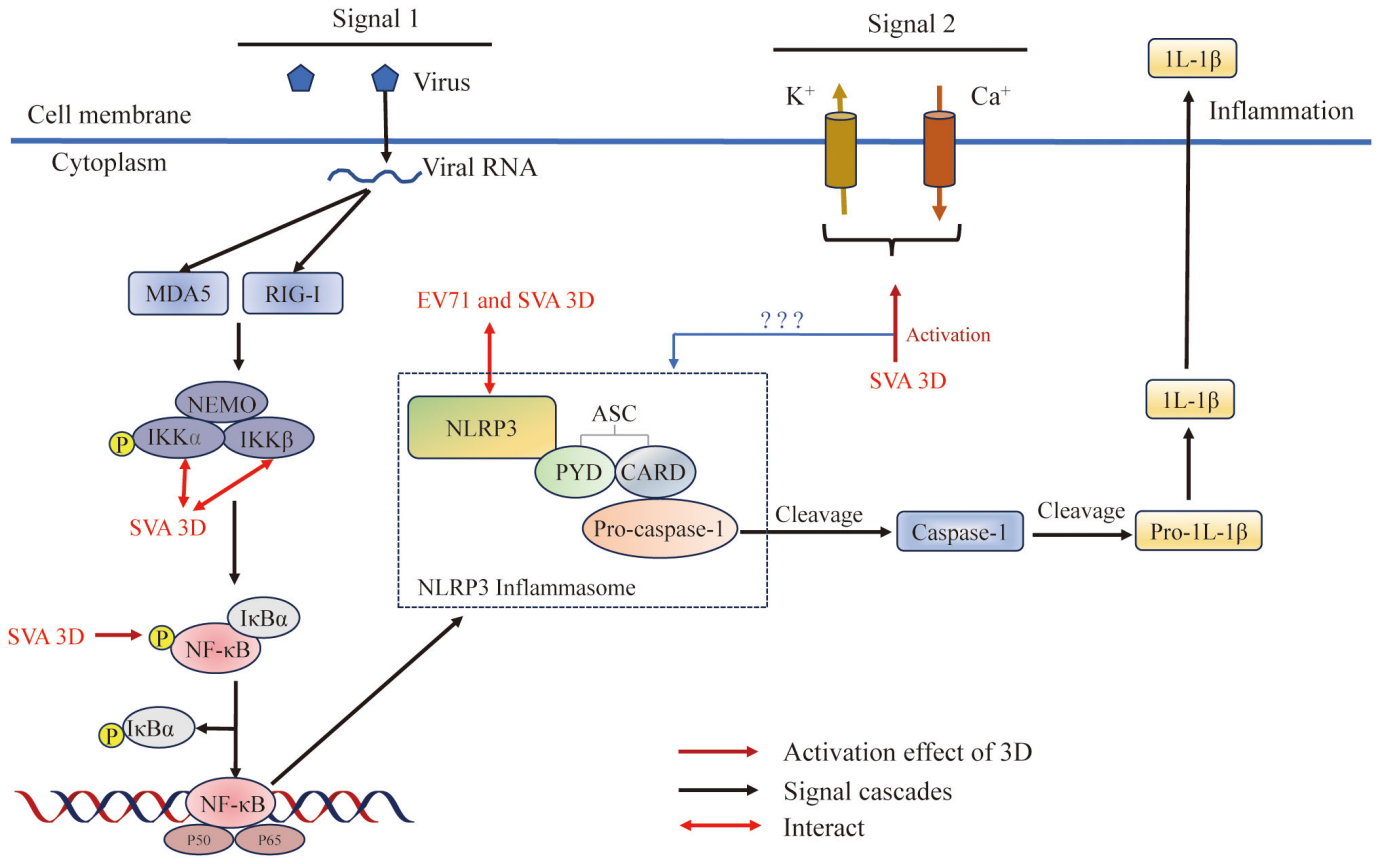
Picornavirus 3D^pol^ regulates inflammasome activation. The NLRP3 inflammasome is an oligomeric complex composed of the NOD-like receptor NLRP3, the adaptor protein ASC, and the effector protein pro-caspase-1 (128). 3D regulates inflammasome activation by inducing NF-κB activation, interacting with NLRP3 to facilitate NLRP3-ASC assembly or inducing calcium influx and potassium efflux. Figure adapted from (129).

Viruses have developed sophisticated mechanisms to manipulate host cellular pathways to facilitate viral replication and evade host defenses. In recent years, an increasing number of researchers have focused on the functions of 3D^pol^ (other than that of a RdRp) during viral infections. 3D^pol^ acts on host cells through interactions with host proteins and plays an important role in inducing cell cycle arrest (**Figure 2A**), regulating host cell translation (**Figure 2B**), inducing apoptosis and autophagy, evading immune responses (**Figure 6**), and activating the NLRP3 inflammasome (**Figure 7**). 3D^pol^ promotes the replication and proliferation of these viruses by regulating these responses.

### 5.1 Induction of cell cycle arrest and regulation of cellular translation

As part of their pathogenic mechanism, many viruses create a favorable environment for viral replication by manipulating the host cell cycle (130–133). The cell cycle is divided into a stationary G_0_ phase, interphase (G_1_, S and G_2_ phases), and a mitotic phase (M phase) (**Figure 2A**). Typically, the cell cycle is controlled by the binding of cyclin-dependent kinases (CDKs) to the corresponding cyclin regulatory subunits (134, 135). Cyclin E/CDK 2 is responsible for regulating cellular S-phase entry from G_1_ (136), cyclin A/CDK 2 regulates S-phase progression by replacing cyclin E (137, 138), and cyclin B/CDK 1 is involved in the mitotic process (139). Previous studies have indicated that the cell cycle affects picornavirus replication (140, 141). Studies using cell cycle inhibitors have revealed that cell cycle arrest at the G_1_ or G_1_/S phase could promote viral replication of CVB3 (142) (**Figure 2A**). In contrast, protein expression and virus yield were significantly reduced after cell cycle arrest in the G_0_ and G_2_/M phases (142). The expression of EV71 3D^pol^ increased the expression of cyclin E and phosphorylated CDK2 T160, which promoted S-phase entry, thereby facilitating viral production (143). In addition, coxsackievirus A16 (CA16)-induced S-phase arrest of the host cell cycle was also observed (143). However, the expression of CVA6 3D^pol^ induced cell cycle arrest in the G_0_/G_1_ phase, which promoted CVA6 replication and viral production (144). Surprisingly, EV71, CA16, and CVA6 all belong to the family *Picornaviridae* and cause hand, foot, and mouth disease; however, there are significant differences. Perhaps these viruses employ different strategies to promote their replication, which leads them to have different characteristics, such as clinical symptoms and epidemiological scopes (144). In addition, EV-D68 3D^pol^ was found to induce cell cycle arrest at the G_0_/G_1_ phase (145), DHAV-1 infection-induced cell cycle arrest in duck embryo fibroblasts (DEFs) in the S phase, and both the S phase and G_0_/G_1_ phase synchronization facilitated the replication of DHAV-1 (146). These results suggest that inducing cell cycle arrest in the S or G_0_/G_1_ phase and promoting viral replication are common strategies for picornaviruses.

Translation of most eukaryotic mRNAs is facilitated by a 5′ cap, a structure absent from picornavirus mRNA, which instead contains an IRES. Cap-dependent translation of cellular proteins is most robust during the G_1_ phase but is impaired at mitosis (142). Picornavirus IRESs evolved to operate in the G_1_ phase, a time at which cap-dependent translation is dominant. Upon infection, picornavirus caused inhibition of the cap-dependent translation machinery and utilized host translation machinery for cap-independent translation of viral proteins mediated by its IRES element within the 5′-UTR (142, 147, 148). Studies have shown that 2A^pro^ and 3C^pro^ cleave eukaryotic initiation factor 4G (eIF4G) (149–151), eukaryotic initiation factor 4A (eIF4A) (152), and eukaryotic initiation factor 5B (eIF5B) (153), leading to host cell translation shutdown. However, it has been reported that EV71 3D^pol^ can enter the cellular nucleus through the NLS to associate with the core splicing factor Prp8 (24) (**Figure 2B**). 3D^pol^ affects the normal function of Prp8 during the second catalytic splicing step, resulting in the inhibition of pre-mRNA splicing and a decrease in the amount of resulting mRNA (24). In contrast to viral proteases blocking host transcription and translation mechanisms, picornaviruses utilize their polymerases to alter cellular gene expression by hijacking the splicing machinery, which potentially providing another advantage for virus replication. Interestingly, another study showed that EV71 3D^pol^ directly increases EV71 IRES-dependent translation as well as cap-dependent translation. 3D^pol^, encoded by EV71, can interact with ribosomal proteins to form complexes with small and large subunits of ribosomes and activate viral and host translation (23) (**Figure 2B**). Since cellular factors known as ITAFs may regulate IRES-mediated translation initiation, 3D^pol^ increases the expression of these cellular proteins by facilitating host translation, favoring viral replication (130, 154). It is conceivable, therefore, that virus-induced cell cycle block may create an environment favorable for viral replication, which could then maximize virus production by manipulating the host cell translation devices.

### 5.2 Regulation of autophagy

Viruses have been shown to employ autophagic machinery to replicate and survive during the infection process (155–157). Recent studies have revealed a distinct mechanism by which EV71 induces apoptosis and autophagy in neural cells (158). EV71 3D^pol^ interacts with the peroxisomal protein acyl-CoA oxidase 1 (ACOX1), attenuates ACOX1 production, and enhances reactive oxygen species (ROS), thereby inducing apoptosis and autophagy in neuronal cells (158). In addition, EMCV 3D^pol^ induces autophagy in BHK-21 cells by activating the ER stress pathway, which ultimately benefits viral replication (159). Furthermore, EMCV-3D^pol^ has been demonstrated to regulate proteins associated with the PERK and ATF6α pathways. Other picornaviruses with similar structures/sequences to EV71 or EMCV 3D^pol^ may also have similar functions; however, further research is needed.

### 5.3 Regulation of the host cellular immune response

The innate immune system is the first line of defense against invading pathogens (160). Upon pathogenic microbial infection, they are recognized by pattern recognition receptors (PRRs), leading to the activation of signaling cascades to generate immune responses (161). Picornaviruses have evolved strategies to evade the innate immune response, and studies have focused mainly on the 2A (26, 162, 163), 2B (164–166), and 3C^pro^ (26, 167). To date, 3D^pol^, essentially known for its significant role in viral genome RNA replication as a polymerase, has been the subject of very few studies concerning its action against the antiviral response. However, previous studies have shown that RdRp can also be involved in regulating innate immune responses (168, 169). The regulatory effect of 3D^pol^ on the host cell immune response mainly manifests as antagonistic effects (**Figure 6**).

#### 5.3.1 3D^pol^ affects RNA sensors

Two cytoplasmic pathogen recognition receptors, melanoma differentiation-associated gene 5 (MDA5) and retinoic acid-inducible gene I (RIG-I), have been identified as sensors for recognizing RNA viruses and stimulating type I IFN expression (170–172). RIG-I recognizes cytoplasmic 5′ triphosphate single-stranded RNA with poly (U/A) motifs and short dsRNA, while MDA5 primarily recognizes long double-stranded RNAs (172–174). LGP2, the smallest member of the RIG-I-like receptors family, is pivotal in regulating the signaling pathway through positive and negative regulation of MDA5 and RIG-I, respectively (175–179). A recent study has shown that cleavage of MDA5 by the 3C^pro^ from Theilovirus leads to dysfunction of MDA5 as an innate immune RNA sensor for IFN induction (180). In addition, FMDV 3C^pro^ inhibits MDA5 protein expression as a mechanism to evade the innate immune response during FMDV infection (181).

RIG-I and MDA5 can sense viral RNA through their C-terminal domains (CTDs), and their caspase activation and recruitment domains (CARDs) can interact with CARDs of the downstream adaptor MAVS to transduce signals (125, 182). Recent studies have shown that EV71 3D^pol^ interacts with CARDs of MDA5 and plays a role in the inhibition of MDA5-mediated beta interferon (IFN-β) promoter activation and mRNA expression (25). This inhibition was also detected by using the RdRp activity knockout mutant (D330A) of EV71 3D^pol^, which demonstrated that EV71 3D^pol^ inhibits IFN-β promoter activity without interfering with viral RNA replication. This study also has shown that CVB3 interacts with MDA5 and downregulates the antiviral signaling initiated by MDA5 (25). In addition, Sarry, Morgan et, al. found that FMDV 3D^pol^ interacts with MDA5 and IFN pathway proteins (IKKα, IKKϵ, IRF3, IRF7, NEMO, and MAVS), which may be responsible for the inhibitory effect on the IFN pathway induction phase by FMDV (127). Moreover, studies have shown that DHAV-1 3CD interacts with RIG-I, interferes with the interaction between RIG-I and MAVS, and degrades RIG-I protein through the proteasomal degradation pathway, thereby inhibiting its mediated antiviral innate immunity to promote DHAV-1 replication (183).

#### 5.3.2 Interference with IFN-mediated signaling

Interferons are cytokines that play a crucial role in regulating and activating the host innate immune response to viral infection and limiting viral replication (126, 184). Upon the production and release of IFNs, the interferon α receptor (IFNAR) is ligated, which subsequently activates Janus-associated kinase 1/2 (Jak1/2) and recruits signal transducers and activators of transcription 1 (STAT1), ultimately leading to the expression of antiviral effector molecules (185–188). Experimental results have shown that EV71 3D^pol^ attenuates IFN-γ-induced tyrosine phosphorylation of STAT1 accompanied by a STAT1 decrease (189); either restoring STAT1 or inhibiting 3D^pol^ activity effectively reversed IFN-γ-induced IRF1 transactivation. However, it is still unknown how the 3D^pol^ regulates STAT1 activation and expression. The specific causes of the decrease in STAT1 transcriptional and/or posttranslational levels by the 3D^pol^ require further investigation.

#### 5.3.3 3D^pol^ targets other proteins associated with the innate immunity response

PGAM family member 5 (PGAM5) can affect the fission/fusion process of mitochondria and inhibit the mitochondrial autophagy pathway (190–193). During EV-D68 replication, the 3D^pol^, via its interaction with PGAM5, can affect the mitochondrial dynamics and suppress the expression of IFN-β by impacting the RIG-I-like receptor signal pathway (27) (**Figure 2**). In addition, extensive studies have shown that the innate immune response and autophagy constitute a mutually coordinated system (194, 195). The autophagy pathway is tightly controlled by numerous autophagy-related genes (ATG) (196–198). Among these, Beclin1 (which encodes BECN1, also called ATG6) is not only a critical regulator in both the early and late steps of autophagy but is also antagonistic to innate immune responses (199–201). It has been reported that EV71 possibly propels 3D^pol^ to interact with Beclin1 in order to regulate the process of autophagy to promote viral replication (202). Further, EV71 3D^pol^ makes use of the interaction with Beclin1 to suppress the type I IFN signaling pathway due to Beclin1 acting as a negative regulator of RIG-I-MAVS mediated IFN response (202, 203). In addition, recent studies have shown that inhibition of IKBKE expression by SERPINB1 induced autophagy to decrease type I interferon signaling, and ultimately promoted SVA proliferation (204). These studies imply the reciprocal coordination between autophagy and innate immunity. However, the mechanism of innate immunity and autophagy regulating viral proliferation and the interaction between these classical pathways remain unclear.

### 5.4 Regulation of the activation of the NLRP3 inflammasome

Inflammasome formation is an innate immune response induced in host cells in response to stimulation by microbial invasion that triggers the maturation of the proinflammatory cytokine interleukin-1β (IL-1β) (205). IL-1β causes the production of cytokines such as IL-6 and TNF-α, and plays a critical role in modulating the immune response during both acute and chronic viral infections (206, 207). IL-1β production is tightly regulated by the NLRP3 inflammasome complex, which consists of the NOD-like receptor NLRP3 and the adaptor protein ASC to recognize danger signals to promote cleavage of the effector protein pro-caspase-1 (128, 208–210). NLRP3 inflammasome activation requires NF-κB activation (priming signal) and assembly of NLRP3-ASC (second signal) (208, 210–212). First, PRRs (such as RIG-I or MDA5) induce a priming signal, which recognize viral nucleic acid and other molecular patterns and then induce NF-κB activation; NF-κB activation acts as a priming signal to initiate the transcription of pro-IL-1β and NLRP3 (210). The second signal is NLRP3-ASC inflammasome assembly, and there are three models for its induction: (i) the ion channel model (213); (ii) the lysosomal rupture model (214); and (iii) the reactive oxygen species (ROS) model (215). The ion channel model, which regulates the concentration of K^+^ or Ca^2+^ in the cells, ultimately helps pathogen-associated molecular patterns (PAMPs) and damage-associated molecular patterns (DAMPs) to enter into the cytosol or cause mitochondrial dysfunction to activate the NLRP3 inflammasome (129), the lysosomal rupture model, which causes the release of cathepsin B after lysosomal damage, leads to NLRP3 activation (216, 217), and the ROS model, which invigorates the circulation of K^+^ and induces NLRP3 inflammasome activation (218, 219).

As reported, SVA can induce IL-1β production (129). SVA has a +ssRNA genome, and it can be recognized by the RIG-I-like receptor of RIG-I/MDA5 and then induce the activation of NF-κB, which leads to the upregulation of NLRP3 and pro-IL-1β transcription (220) (**Figure 7**). Meanwhile, SVA 3D^pol^ promotes the activation of NF-κB by interacting IKKα and IKKβ, which upregulates the NLRP3 and pro-IL-1β transcription (129). These results suggested that the effects of SVA RNA and 3D^pol^ induction of NF-κB activation are superimposed. This study also proved that SVA 3D^pol^ directly interacts with the NATCH domain of NLRP3 through the N-terminus (amino acids 1 to 154) to facilitate NLRP3-ASC assembly, which induces IL-1β production (129). At the same time, 3D^pol^ also affects the production of IL-1β through ion channels. 3D^pol^ induces calcium influx and potassium efflux to activate the NLRP3 inflammasome at the second signaling step (129, 221). In addition, other studies revealed a novel mechanism by which EV71 stimulates the activation of NLRP3 inflammasome by the virus-encoded 3D^pol^. 3D^pol^ interacts directly with NLRP3 to facilitate the assembly of NLRP3 inflammasome complex by forming a “3D-NLRP3-ASC” ring-like structure (222). These studies revealed a new role of picornavirus 3D^pol^ as an important regulator of inflammatory responses and provided new insights into the development of drugs for the treatment and prevention of virus-associated inflammation and diseases.

## 6 Conclusions

The past decade has been fruitful for the viral RdRp structure field, and providing insights into the initiation of RNA synthesis and the replication elongation processes in picornavirus (21, 36, 92). However, the NLS sequence carried by picornavirus 3D^pol^, combined with its ability to interact with other viral proteins, viral RNA and cellular proteins, indicate that the noncatalytic role of picornavirus 3D^pol^ could be underestimated. In addition to its traditional role in replication, 3D^pol^ can interact with several host proteins, which participate in a variety of biological processes in host cells, such as cell cycle progression, protein synthesis, apoptosis and autophagy, and these interactions may result in multiple consequences that benefit the viruses in different lifecycle stages. Interactome analysis has been widely applied to explore virus–host interactions. Yeast-two-hybrid assays and proteomic approaches based on MALDI-TOF mass spectrometry have been used to screen host factors that may interact with viral proteins in infected cells (24, 123, 223, 224). Advanced approaches using immunoprecipitation coupled with liquid chromatography−tandem mass spectrometry (LC−MS/MS) can be practical to broadly detect cellular proteins that associate with viral proteins (23, 225). Further development of these related technologies and methods may help to identify and validate novel host proteins that interact with 3D^pol^ and provide a better understanding of how 3D^pol^ regulates and usurps host processes, while also helping to uncover the mechanisms underlying pathogenesis.

Importantly, there are currently only limited therapies for the treatment of picornavirus infection. The key role of 3D^pol^ in viral replication and its structural and sequence conservation make it a promising target for specific antiviral therapeutics (19, 31, 226). Several compounds that bind to 3D^pol^ active sites to block viral replication have been identified, which markedly reduce the synthesis of viral RNA by interacting with or occupying the 3D^pol^ active sites to inhibit enzyme function (227–229). Therefore, further elucidating the structures and molecular functions of 3D^pol^ is valuable and could be useful for future antiviral treatment of picornaviruses.

## Author contributions

CX: Formal analysis, Investigation, Writing – original draft. MW: Formal analysis, Supervision, Writing – review & editing. AC: Funding acquisition, Supervision, Writing – review & editing. QY: Formal analysis, Supervision, Writing – review & editing. JH: Formal analysis, Supervision, Writing – review & editing. XO: Formal analysis, Supervision, Writing – review & editing. DS: Formal analysis, Supervision, Writing – review & editing. YH: Formal analysis, Supervision, Writing – review & editing. ZW: Formal analysis, Supervision, Writing – review & editing. YW: Formal analysis, Supervision, Writing – review & editing. SZ: Formal analysis, Supervision, Writing – review & editing. BT: Formal analysis, Supervision, Writing – review & editing. XZ: Formal analysis, Supervision, Writing – review & editing. ML: Formal analysis, Supervision, Writing – review & editing. DZ: Formal analysis, Supervision, Writing – review & editing. RJ: Formal analysis, Supervision, Writing – review & editing. SC: Formal analysis, Supervision, Writing – review & editing.
